# Trust in relationships: a preliminary investigation of the influence of parental divorce, breakup experiences, adult attachment style, and close relationship beliefs on dyadic trust

**DOI:** 10.3389/fpsyg.2023.1260480

**Published:** 2023-11-02

**Authors:** Ceren D. Yılmaz, Timo Lajunen, Mark J. M. Sullman

**Affiliations:** ^1^Department of Psychology, Near East University, Nicosia, Cyprus; ^2^Department of Psychology, Norwegian University of Science and Technology, Trondheim, Norway; ^3^Department of Social Sciences, School of Humanities and Social Sciences, University of Nicosia, Nicosia, Cyprus

**Keywords:** romantic relationships, attachment styles, close relationship beliefs, dyadic trust, mediation

## Abstract

**Introduction:**

Trust is essential for establishing stable and fulfilling romantic relationships between partners. Development of trust, however, can be assumed to depend on many factors related to an individual's earlier experiences and relationship-related beliefs. This study aimed to investigate how adult attachment style (anxious, avoidant), experiences about parents' divorce and breakdown of one's own romantic relationship, and relationship beliefs are related to the level of dyadic trust in romantic relationships.

**Methods:**

The present study included 131 Turkish undergraduate university students (55.7% women) from different faculties. The research instrument had questions about parents' and respondents' own relationship status, Dyadic Trust Scale (DTS), Experiences in Close Relationships Inventory-Revised (ECR-R), and Inventory of Close Relationship Beliefs (ICRB), in addition to background questions. The data were analyzed using descriptive statistics, *t-*tests, Pearson correlations, regression analyses and mediation analyses.

**Results:**

Respondents whose parents had divorced or who had experienced a relationship breakdown had lower dyadic trust scores than those without these experiences. The trust scores correlated negatively with anxious and avoidant attachment styles and positively with relationship belief scales, although the correlations to “external factors” were not statistically significant. In regression analysis, anxious and avoidant attachment styles explained 42% and relationship beliefs 25% of the variance in trust. The only significant predictor among beliefs was “individuality.” Mediation analysis showed that the effects of anxious attachment style on trust were fully mediated by the relationship belief in “individuality.” The avoidant attachment style had a direct relationship to trust.

**Discussion:**

The results show that anxious attachment style influences trust via relationship beliefs, while avoidant attachment style has a strong direct effect on trust as well as weaker effects via beliefs. The results are discussed in the context of Turkish culture and horizontal collectivism.

## 1. Introduction

Trust is a key factor in successful romantic relationships. Trust evolves throughout the various stages of dating, flirting, engagement, and marriage (Aron et al., 1995), encompassing concepts such as intimacy, attachment, self-respect, and love. The crucial role of trust in romantic relationships has been reported particularly during adolescence and young adulthood, influenced by various personal and relational factors, including the attachment style (Mikulincer and Shaver, 2007; Kim et al., 2017). Trust plays a pivotal role in maintaining functional and fulfilling romantic relationships, deepening intimacy, and providing continuity (Larzelere and Huston, 1980; Kemer et al., 2016). Lack of trust can lead to negative reactions, lying, low relationship quality perception, and attachment anxiety, negatively impacting relationships (Simpson, 2007; Campbell et al., 2010; Uysal et al., 2012; Laborde et al., 2014; Towner et al., 2015; Gabbay and Lafontaine, 2020). On the other hand, trust positively affects relationship satisfaction in close romantic relationships (Büyükşahin and Hovardaoglu, 2007).

Baldwin et al. (1996) found attachment-style differences in response to trusting a partner and, thus, showed that the attachment style of an individual might be related to the trust they have in their partner. This is understandable because attachment styles, as explained, significantly influence the formation of trust bonds within romantic relationships (Ainsworth, 1991). These attachment styles are shaped by an individual's psychological development, maturity, and mental wellbeing (Carter et al., 2013). Research on attachment styles has revealed that securely attached individuals are more comfortable and facilitating in the early stages of relationships, while anxious individuals fear rejection and avoidant individuals distance themselves (Mikulincer and Shaver, 2007). Secure individuals expect a more positive response from a trusted partner compared to insecure individuals (Baldwin et al., 1996). Avoidant attachment style is associated with individuals who struggle to develop trust and exhibit less effort and discomfort in close relationships. Conversely, anxiety is linked to individuals who experience anxiety about rejection, often leading to short-lived relationships (Carter et al., 2013). Anxious individuals struggle to maintain trust and fear rejection or abandonment, while avoidant individuals have difficulty establishing intimate relationships (Hazan and Shaver, 1987; Feeney and Noller, 1990; Mikulincer and Erev, 1991; Brennan and Shaver, 1995). However, some researchers argue that individuals with anxious attachment may show greater love and commitment due to seeking reassurance (Duemmler and Kobak, 2001). In summary, individuals with anxious attachment tend to enter relationships quickly but struggle to maintain them, while those with avoidant attachment generally engage in short-lived relationships due to lower commitment and trust (Ehrenberg et al., 2012).

Close relationship beliefs play a pivotal role in romantic relationships as they are influenced by past experiences, memories, and cognitive structures. Fletcher and Kininmonth (1992) developed the Relationship Belief Scale (RBS) to measure the dimensions of intimacy, individuality, passion, and external factors that shape romantic beliefs and contribute to the development of trust in the success of long-term close relationships. “Intimacy” focuses on beliefs concerning interpersonal attitudes and interactions related to the development of intimacy and closeness. The second factor, “External Factors,” includes beliefs related to the importance of external factors or problems. The third factor, “Passion,” contains topics related to sex and vitality. The fourth factor, “Individuality,” combined independence and equity (Fletcher and Kininmonth, 1992). Within these four main factors, the RBS measures 18 different facets reflecting different aspects of relationships (e.g., respect, love, children, gender, and equity). The RBS has been used earlier in cross-cultural settings, showing that Chinese (from Taiwan) respondents prioritized ideals denoting financial resources and extended family to a greater extent than European Americans (Lam et al., 2016). It can be assumed that relationship beliefs are closely related to trust because beliefs are used to form expectations and often unwritten norms for the behavior of the partner or spouse.

It can be assumed that relationships between people, such as friendship networks, family relations, and romantic relationships, reflect cultural values. The present study was conducted in Turkey, which is characterized by “horizontal” collectivism rather than “vertical collectivism” or Western individualism. Vertical collectivism is characterized by a sense of service and sacrifice for the in-group and an acceptance of the benefits of inequality and rank, while the horizontal dimension includes a sense of social cohesion and oneness with members of the in-group and a valuation of similarity on most attributes across individuals, especially on status (Singelis et al., 1995; Çukur et al., 2004). The three-generation study among Turkish grandmothers, mothers, and grandchildren by Kagitçibaşi and Ataca (2005) showed that Turkish families desire close relations rather than individualistic separation and that this Turkish “autonomous-related self” is different from both the (autonomous) separate self typical to the Western individualistic family pattern and the (heteronomous) related self typical to the traditional collectivistic family pattern. This “autonomous-related self” typical of contemporary Turkish culture can be reflected in beliefs and expectations related to romantic relationships. It should be noted that Turkey has undergone a rapid change from a rural collectivistic society to a more urban and individualistic one (Kagitçibaşi and Ataca, 2005), which might be reflected in relationship beliefs among men and women. For instance, Kemer et al. (2016) found that married Turkish men were more emotionally jealous than women. Those who distrusted their partners displayed heightened cognitive jealousy and behavioral reactions, potentially leading to controlling behaviors. This control could manifest in the form of restrictions placed on a wife or girlfriend. Hence, it's plausible that such tendencies might impact how Turkish individuals perceive the “Individuality” aspect of the RBS.

In addition to attachment style and beliefs related to close relationships, previous experiences of relationship breakdown or parents' divorce might influence the level of trust the young adults experience in their relationships (Roth et al., 2014). Earlier research shows that women who have experienced parental divorce in childhood or adolescence tend to distrust others (Størksen et al., 2006; Oldehinkel et al., 2008; Viršilaite and Bukšnyte-Marmiene, 2021). In the present study, young adults' experiences with relationship breakdown and parents' divorce history were measured.

Given that heterosexual romantic relationships are based on a sexual relationship between different genders, we could assume that it is necessary to investigate gender differences in adult attachment, relationship beliefs and dyadic trust, although exact gender differences could not be hypothesized for all study variables and for this sample. While the classic attachment theory that focuses on children does not assume gender differences in attachment style (Del Giudice, 2019), a meta-analysis of gender differences in adult romantic attachment reported higher avoidance and lower anxiety for men than for women. Although these differences varied across geographic regions, the largest gender differences were observed in Europe and the Middle East (Del Giudice, 2011, 2019). Moreover, earlier studies have highlighted gender differences in dyadic trust (Çetinkaya et al., 2008; Kemer et al., 2016) and relationship beliefs (Frazier and Esterly, 1990). In the present study, the gender differences were tested, and gender included in analyses when possible.

The aim of the study was to investigate how adult attachment style (anxious, avoidant), experiences about parents' divorce and breakdown of one's own romantic relationship, and relationship beliefs influence the level of dyadic trust in romantic relationships. Since heterosexual romantic relationships are very much based on sex and gender roles, we expected that there might be differences between men and women in relationships between attachment, relationship beliefs and dyadic trust. We hypothesized the following relationships:

1. Participants who had experienced parental divorce or had separated themselves from a close relationship would score lower in trust than those whose parents are married or who have not experienced a breakdown of a romantic relationship.
2. An anxious and avoidant attachment style would have a negative relationship with interpersonal trust.
3. Positive relationship beliefs would have a positive relationship with interpersonal trust.
4. The effects of attachment style would be at least partly mediated by relationship beliefs, i.e., attachment style would influence the beliefs, which, in turn, would be related to interpersonal trust.

## 2. Method

### 2.1. Participants and procedure

The sample size estimation was conducted with G^*^power (effect size = 0.30; power = 0.95; one-tailed). The estimated sample size was n=111. The sample consisted of 131 undergraduate students of various majors (mean age = 21.64, SD = 1.93), of whom 55.7% were women. The participants were student volunteers who completed a 20-min survey during their class hour. The participants did not receive any benefit from participating in the study. Participants were informed of their rights to voluntary participation and the option to stop answering at any time.

Ethical approval was obtained from the Near East University Ethical Committee and the University of Kyrenia Ethical Committee (protocol number: YDU/SB/2020/615).

### 2.2. Instruments

#### 2.2.1. Demographic information form

The demographic information form included questions about gender (woman, man), age (full years), relationship status of the parents (married with each other, divorced, or living separately, widowed, or single) and relationship status of the respondent (in a relationship, separated, not having had a romantic relationship). Most parents (*n* = 105, 80.2%) were married to each other, 21 (16.0%) were divorced or living separately, and two (1.5%) were widows or single parents not having been married. Since the number of widows or single parents was low, they were excluded from the analysis related to parental relationship status. Most participants reported being in a romantic relationship (*n* = 64, 48.9%), 46 (35.1%) reported being single because of a breakdown of a relationship, and 21 (16.0%) reported never having been in a romantic relationship.

#### 2.2.2. Dyadic Trust Scale (DTS)

The Dyadic Trust Scale (Larzelere and Huston, 1980) is a one-dimensional seven-point scale (response alternatives ranging from “never” to “always”) used to assess trust in marriage and romantic relationships. A high score in DTS indicates high trust in the relationship. The Turkish translation by Çetinkaya et al. (2008) was applied in the present study. While the original DTS contains eight items, the 6th item was excluded from the Turkish scale because of low item loading in the adaptation study, resulting in a 7-item scale (Çetinkaya et al., 2008). The alpha coefficient for the scale was 0.94.

#### 2.2.3. Experiences in Close Relationships Inventory-Revised (ECR-R)

The Experiences in Close Relationships Inventory-Revised (ECR-R), developed by Fraley and Shaver (2000), measures anxious and avoidant attachment styles. The respondents evaluate the statements with a seven-point Likert scale, with response alternatives varying from “do not agree at all” (1) to “totally agree” (7). The scale was translated into Turkish and validated in Turkey by Selçuk et al. (2005). A high score denotes a high level of anxious or avoidant attachment style. The alpha coefficients were 0.87 and 0.91 for anxious and avoidant attachment styles, respectively.

#### 2.2.4. Inventory of Close Relationship Beliefs (ICRB)

The Inventory of Close Relationship Beliefs, developed by Fletcher and Kininmonth (1992), measures beliefs associated with a successful close relationship. The ICRB was translated into Turkish and adapted to the Turkish population by Öztekin (2016). The scale includes statements (six-point response scale) related to 18 different aspects of a good relationship (e.g., respect, support, personal security, gender, independence). These 18 facets form sub-scales of intimacy, external factors, passion, and individuality. The alpha reliability coefficients were 0.90, 0.75, 0.79, and 0.74, thus indicating sufficient internal consistency.

### 2.3. Data analysis

IBM SPSS 28.0 was used for calculating descriptive statistics, *t-*tests, reliability statistics, correlations and regression analyses. JASP was used for mediation analysis.

## 3. Results

### 3.1. Descriptive statistics of the study variables and mean differences between men and women

Since gender is an important factor in adult heterosexual relationships, gender differences were calculated for the two attachment styles, the four relationship belief scales, and the dyadic trust scores. The tests of gender differences were considered exploratory and therefore direction of gender differences was not specified; consequently, two-tailed *t-*tests were used. The only hypothesis (H1) was that a gender difference occurs in the variable concerned.

The descriptive statistics separately for men and women and *t*-tests for gender difference are presented in Table 1. Table 1 lists the means (M) and standard deviations (SD) for men and women on study variables, as well as the independent means *t-*test values for gender differences. Men scored higher than women in anxious attachment style, while women scored higher on the individuality scale of the ICRB. Women seem to value individuality (independence, equity) in relationships more than men. No gender difference was found in the other variables.

**Table 1:** Descriptive statistics and *t-*tests.

	**Men**		**Women**			
**Variable**	**M**	**SD**	**M**	**SD**	***t*** **-test**	**Cohen's d**
Trust	5.47	1.48	5.52	1.32	−0.20	0.04
Anxiety	3.99	1.21	3.61	1.02	1.99^*^	−0.35
Avoidance	2.89	1.18	2.87	1.09	0.10	−0.02
Passion	4.59	0.98	4.32	1.05	1.54	−0.27
Individuality	4.47	0.89	5.19	0.73	−5.07^**^	0.89
Intimacy	4.87	0.72	4.95	0.57	−0.75	0.13
External factors	4.03	0.69	3.89	0.71	1.15	−0.20

^*^p < 0.05; ^**^p < 0.001. df = 129.

### 3.2. Parental divorce, breakdown of one's own relationship and interpersonal trust (hypothesis 1)

An independent samples *t-*test was employed to investigate the mean difference in trust between respondents with parents who had divorced or separated and those with parents who remained together. Respondents with married parents scored higher (M = 5.65, SD = 1.86) on the Dyadic Trust Scale than those with divorced parents (M = 4.54, SD = 1.98), t_(22.93)_ = 2.46, *p* = 0.011, Cohen's d = 0.82. This suggests that experiencing parental divorce might be associated with reduced trust in relationships. However, it's essential to note that the sample size for respondents with divorced parents was small (*n* = 21), which limits the generalizability of the results. These results confirmed Hypothesis 1, that respondents having experienced parental divorce experienced less trust in relationships.

In addition to parental divorce, respondents also provided information about their current relationship status, choosing from the options: no relationship, relationship ended, or in an ongoing romantic relationship. A one-way ANOVA revealed a statistically significant main effect of relationship status, F_(2, 130)_ = 9.82, *p* < 0.001, η^2^ = 0.13. Bonferroni corrected pairwise comparisons indicated that respondents in an ongoing relationship had higher trust scores (M = 6.01, SD = 1.03) than both those who had ended a relationship (M = 4.96, SD = 1.64), *p* < 0.001, and those who had never been in a romantic relationship (M = 5.10, SD = 1.19), *p* = 0.019. There was no statistically significant difference in trust scores between respondents who had ended their relationship and those who had never been in one. These results suggest that positive experiences in a romantic relationship may bolster interpersonal trust. Moreover, it can be inferred that respondents who had never been in a romantic relationship based their trust responses on their beliefs about romantic relationships in general. These results confirmed Hypothesis 1, which posited that respondents who had experienced a relationship breakdown would have less trust in relationships. No hypothesis was formed about not having been in a romantic relationship and trust.

### 3.3. Correlations between attachment styles, relationships beliefs and trust (hypotheses 2 and 3)

Correlations among study variables are displayed in Table 2. Age had significant negative correlations with anxious and avoidant attachment style and a positive correlation with the passion scale of the ICRB. Male gender correlated positively with anxious attachment style and individuality scale of the ICRB. Trust correlated negatively with anxiety and avoidance but positively with passion, individuality, and intimacy but not with external factors scale of the ICRB. In general, ICTRB scales correlated negatively with anxious and avoidant attachment styles. These findings confirmed hypotheses 2 (negative relationship between anxious and avoidant attachment style and trust) and 3 (positive relationship between positive relationship beliefs and trust).

**Table 2:** Correlations between study variables.

	**1**	**2**	**3**	**4**	**5**	**6**	**7**	**8**
1. Age	1.00							
2. Gender	0.09	1.00						
3. Trust	0.16	−0.02	1.00					
4. Anxiety	−0.20^*^	0.17^*^	−0.47^***^	1.00				
5. Avoidance	−0.23^**^	0.01	−0.62^***^	0.51^**^	1.00			
6. Passion	0.18^*^	0.13	0.31^***^	−0.14	−0.48^***^	1.00		
7. Individuality	0.08	−0.41^***^	0.45^***^	−0.41^**^	−0.39^**^	0.28^**^	1.00	
8. Intimacy	0.15	−0.07	0.38^***^	−0.16	−0.56^**^	0.55^***^	0.55^***^	1.00
9. External factors	0.06	0.10	0.16	0.13	−0.12	0.51^***^	0.07	0.42^***^

^*^p < 0.05; ^**^p < 0.01; ^***^p < 0.001.

### 3.4. Mediation effects of individuality on attachment—trust relationship (hypothesis 4)

**Figure 1:**
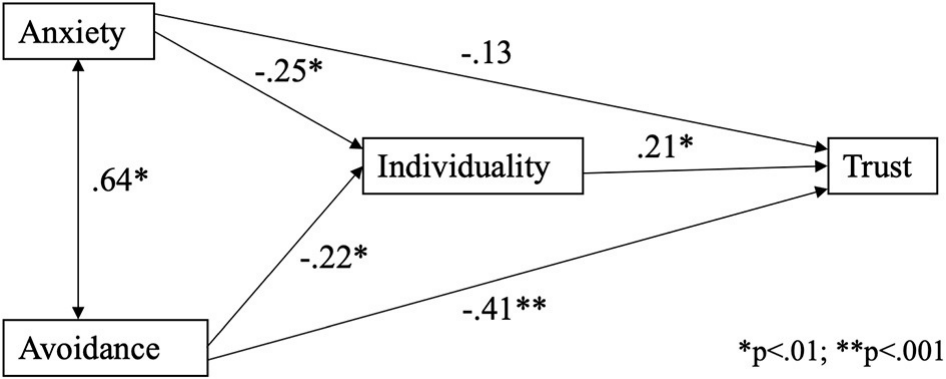
Attachment style, relationship beliefs, and dyadic trust: the mediation model.

Hypothesis 4 proposed that the effects of attachment style on dyadic trust would be at least partly mediated by relationship beliefs. Consequently, Figure 1 describes a mediation model in which individuality was assumed to mediate the relationship between attachment styles and interpersonal trust. This model assumed that attachment style influences both the development of close relationship beliefs and interpersonal trust. Since the regression analysis results (Table 3) showed that only the individuality beliefs were statistically significantly related to trust, only individuality was included in the final mediation analysis as the mediator. Mediator analyses with the other three RBS scales (intimacy, external factors, passion) as mediators were conducted, too, but no significant relationship between the mediator and trust was found. JASP mediation analysis (Figure 1) showed a statistically significant (*p* < 0.001) direct effect of avoidant attachment style on trust, whereas the direct effect of anxious attachment style on trust was not statistically significant (*p* = 0.054). The indirect effect of anxiety on trust via individuality was statistically significant (estimate = −0.05, z = −2.13, *p* = 0.033). Similarly, the indirect effect of avoidance on trust via individualism was statistically significant (estimate = −0.05, z = −2.10, *p* = 0.046). The total effects of both anxiety (estimate = −0.19, z = −2.70, *p* = 0.007) and avoidance (estimate = −0.46, z = −6.68, *p* < 0.001) on trust were statistically significant. The model explained 46% of the variance in trust scores.

**Table 3:** Hierarchical regression analysis predicting trust scores.

**Model**	**Variable**	**B**	**Std. error**	**Beta**	**t**	**CI95%**	
1	Passion	0.20	0.14	0.15	1.48	−0.07	0.47
	Individuality	0.58	0.15	0.36	3.85^**^	0.28	0.87
	Intimacy	0.19	0.24	0.09	0.80	−0.28	0.67
	External factors	0.06	0.19	0.03	0.30	−0.31	0.42
2	Anxiety	−0.26	0.10	−0.21	−2.67^*^	−0.45	−0.07
	Avoidance	−0.64	0.10	−0.52	−6.60^**^	−0.83	−0.44

^*^p < 0.01; ^**^p < 0.001.

The mediation model results show that the effects of anxious attachment on trust were fully mediated by individuality, whereas the direct effect of avoidant attachment style on trust was stronger than the mediation effect of individuality. We can, therefore, conclude that Hypothesis 4 was confirmed.

When evaluating the results of the mediation analysis, it should be borne in mind that these analyses are based on a theoretical model, and no causal relationships can be confirmed based on cross-sectional data.

## 4. Discussion

This study aimed to investigate how attachment style (anxious, avoidant), experiences about parents' divorce and breakdown of one's own romantic relationship, and relationship beliefs are related to the level of dyadic trust in romantic relationships. The findings were consistent with previous research showing that experiencing one's own relationship breakdown (Roth et al., 2014) or parental divorce or separation can reduce dyadic trust in relationships (Størksen et al., 2006; Oldehinkel et al., 2008; Viršilaite and Bukšnyte-Marmiene, 2021). Similarly, our findings among Turkish students highlighted the significance of relationship beliefs (intimacy, individuality, passion, and external factors) and attachment styles (avoidant, secure) in dyadic trust (Campbell and Stanton, 2019). A recent meta-analysis of 53 articles revealed that both anxious and avoidant attachment dimensions were negatively, concurrently, and longitudinally associated with interpersonal trust (Bao et al., 2022). While a person's own attachment style seems to influence the trust felt in relationships, the partner's attachment style can have an impact on trust, too. Notably, while an individual's attachment style can shape trust in relationships, their partner's attachment style can also exert influence on trust experienced in a relationship. Kane et al. (2007) demonstrated this dynamic in a study of 305 couples, finding that men were less satisfied when their female partners exhibited higher attachment anxiety, and women were less satisfied when their male partners displayed increased avoidance (Kane et al., 2007). Unfortunately, our study focused solely on one's own attachment style, and therefore, we did not measure experiences related to the partner's attachment style.

Gender did not play a significant role in trust scores, although previous research has reported higher trust scores among men than women (Çetinkaya et al., 2008). While no gender difference was found in the level of trust in the present study, men scored lower than women in their belief in individuality in relationships and higher in anxious attachment style. As the mediation model shows, anxious attachment style was related to individuality, which, in turn, was related to lower trust. If Turkish men are more prone to have an anxious attachment style than women, they can be expected to value individuality less in relationships, which would have a negative effect on trust. The difference between men and women in emphasizing individuality might reflect the difference between traditional and (post)modern views of romantic relationships, which can be observed in Turkish society. Women may place greater emphasis on individuality (i.e., independence and equity) in romantic relationships compared to men due to the potential imposition of traditional female gender roles on women.

In the present study, age groups did not differ significantly in trust scores, which might be related to a relatively small variance in the age of the respondents. In their study among 34 couples, Norona et al. (2017) did not find any age effect on trust level. However, trust scores were significantly lower among students with divorced or separated parents, indicating the influence of parental relationships (King, 2002). In addition, trust scores varied significantly based on personal experiences related to romantic relationships, aligning with Larzelere and Huston (1980), who reported higher trust scores in long than short relationships. These findings show that experiences of relationships breaking down because of either parental divorce or the end of one's own romantic relationship can reduce the experienced trust. These negative experiences are lived through examples of the vulnerability inherent in romantic relationships. Interestingly, participants who had never been in a romantic relationship scored lower in trust compared to those in ongoing romantic relationships. However, their trust scores did not differ from those who had ended a relationship. This suggests that dyadic trust and positive beliefs about romantic relationships develop over time within the context of a trustworthy relationship. It's also possible that attachment insecurities have a stronger impact on trust beliefs in individuals without romantic relationship experience compared to those who have built trust in past or present relationships. Hence, attachment style may influence both the expectations and beliefs before entering a romantic relationship as well as the level of trust experienced within a relationship while positive experiences about trust may alleviate the effects of attachment insecurities.

Correlation and regression analysis results showed that both anxious and avoidant attachment styles were negatively related to dyadic trust, which is in line with earlier results by Mikulincer (1998), Kim et al. (2017), and (Bao et al., 2022). The negative effect of anxious and avoidant attachment styles on trust is understandable: a person with an anxious attachment style does not trust that the relationship continues, while a person with an avoidant attachment style keeps a distance from the romantic partner and, thus, does not let the interpersonal trust develop. Trust means confidence in the continuation of the relationship and willingness to share one's feelings with one's romantic partner. Positive correlations were found between trust scores and relationship belief factors, individuality, intimacy, passion, and external factors, which confirms the early findings of Fletcher et al. (1994). This could be expected because all four belief scales measure positive beliefs related to relationships. People having positive beliefs about relationships are obviously readier to trust their partners than people with negative beliefs.

Anxiety attachment showed a negative correlation with close relationship belief scores except with external factors, which was also reported by Stackert and Bursik (2003). Similarly, as in Hadden et al. (2014), avoidant attachment style showed a negative correlation with all four close relationship beliefs, although the correlation to external factors was not statistically significant. These correlations show the distinct character of the external factors scale. The external factors include such facets as personal security, important others, finance, commonality, and children, i.e., the material and practical aspects of a close relationship. Other aspects of relationship beliefs tangle with more emotional and personal aspects, such as passion, intimacy, and individuality. It is understandable, therefore, that the attachment style has a stronger relationship to those three more emotional beliefs. In the regression analysis, only individuality appeared as a significant predictor of trust, which is partly due to intercorrelations among the four belief scales. Individuality as the only predictor of trust might be specific to the close relationships in the Turkish context. Turkish culture is characterized by horizontal collectivism, in which the self is perceived as an equal member of the collective, such as the family (Singelis et al., 1995; Çukur et al., 2004). In the Turkish family context, the families of both spouses often intervene in the couple's life, and an individual's wishes might not be respected as much as in individualistic countries in which personal autonomy and independence are emphasized. The positive relationship between individuality as a relationship belief and trust means that young Turkish educated students see that respect for equity in marriage or in a relationship indicates trust.

While the sample size was too small to conduct separate path analyses for men and women, the results showed that the only relationship belief dimension correlated with being female was individuality. Mean comparisons showed that men scored lower in individuality than women. These findings suggest that young, educated Turkish men and women have differing perceptions regarding the importance of independence. A large study conducted among students in 16 universities in Turkey revealed that gender plays a more potent role in predicting attitudes toward women than does the degree of masculinity-femininity. Participants from politically conservative regions, as well as those with a pronounced inclination toward vertical collectivism (characterized by societal hierarchy and inequality), demonstrated more conventional perspectives compared to their counterparts from less conservative locales and those with less vertical collectivism tendencies (Bugday et al., 2021). Furthermore, the influence of vertical collectivism on attitudes toward women was markedly more pronounced among male participants than among females (Bugday et al., 2021). In another study involving Turkish university students, significant gender differences were observed in perceptions related to honor killings of women. Turkish men tended to attribute less responsibility to the assailant and suggested milder punishments compared to Turkish women. Conversely, Turkish women assigned less responsibility to the victim in instances of alleged adultery than did their male counterparts (Caffaro et al., 2014). These studies, including our own, suggest that a woman's independence and her perceived equality with her spouse might lead to disagreements and potentially reduce trust in romantic relationships. Whereas Turkish men tend to uphold more traditional roles for women, Turkish women are generally more inclined to expect equality between spouses.

The mediation model indicated that the path from anxious attachment style to trust was fully mediated by individuality, while the direct relationship from avoidant attachment style to trust was stronger than the mediated relationship. An anxious attachment style reduces the belief in individuality and, hence, leads to lower trust. It seems that people with anxious attachment styles perceive a romantic partner's need for independence as a threat to the relationship. Avoidance is directly related to lower trust because, for an avoidant person, trust is simply not important in the relationship. In this manner, the mediation model illustrates two distinct pathways through which attachment style is related to trust.

The study has some limitations. Firstly, it was based on volunteer participation. This might lead to self-selection bias, whereby participants scoring high in avoidant attachment style might also avoid participating. However, since the study was conducted during class hours and not online, the potential for self-selection bias should be less than in internet-based studies. Moreover, the issue of self-selection is inherent in all attachment studies based on self-reports, as participation in psychological studies must always be voluntary. In addition to possible self-selection, it should be noted that attachment in adult romantic relationships might be lower that the attachment theory suggests (Fraley et al., 2011), which would lead to less stable relationships between attachment, relationship beliefs and trust. If the attachment style can change within time and in context, also the relationships between attachment style and relationship outcomes could vary. The second shortcoming relates to the small sample size. While the sample size was deemed sufficient when estimated with a sample size calculator, much larger samples are necessary for more complex sub-group analyses. The results indicated that relationship-related independence beliefs particularly divided men and women and potentially highlighted one of the most crucial factors in romantic relationships among Turkish couples, namely, beliefs related to a woman's role in the relationship. Disagreements about a wife's or girlfriend's equality with her spouse or partner might be among the primary challenges in Turkish romantic relationships, leading to a lack of dyadic trust, relationship breakdown, and, in extreme cases, violence. Unfortunately, the small sample size, resulting from the data collection strategy (paper-and-pencil questionnaires distributed during lectures) and a lack of resources, prevents separate analyses and structural equation modeling for men and women. The mediation model should be tested separately for both genders and different groups based on relationship status. In addition, other mediator models than relationship beliefs could be tested. It is also important to bear in mind that such causal models as mediator models are always based on theoretical assumptions in cross-sectional studies such as ours. True causality can be established only in experimental or follow-up studies, which, on the other hand, are not feasible when studying this topic. Thirdly, the generalizability of the results is limited not only by the small sample size but also by the sample characteristics. The sample was comprised of young, educated university students who do not fully represent the Turkish population, even though a carefully collected non-internet-based classroom sample might closely resemble Turkish students. Given that more liberal views are typical among educated youth, future studies should include participants whose education is restricted to obligatory schooling, i.e., 8 years. Finally, the findings might predominantly represent contemporary perspectives of young, educated Turkish adults. Therefore, additional research in more collectivistic and individualistic cultures is necessary to further explore the role of relationship beliefs as mediators between adult attachment style and dyadic trust.

## 5. Implications to further research and practice

This study offers a preliminary examination of the mechanisms by which anxious and avoidant attachment styles may influence dyadic trust through relationship beliefs. It is noteworthy that beliefs about independence seem to play a pivotal role in dyadic trust, with these beliefs holding different degrees of importance for men and women. This observation, which might be particularly relevant for Turkish and other semi-collectivistic cultures, has profound implications for couple therapists and counselors. Divergent views between spouses regarding a woman's role and equality within relationships can lead to reduced trust, heightened emotional and behavioral jealousy, and, tragically, to relationship breakdowns and instances of domestic violence. As such, our initial findings should catalyze further extensive research and alert family therapists and couple counselors to this issue. If independence proves to be a cornerstone in building dyadic trust, it is imperative to communicate this insight to the broader public. Fostering mutual understanding between partners about a woman's role in marriage might enhance trust and overall relationship quality, especially in societies where collectivistic perspectives on marriage and relationships prevail.

## Data availability statement

The raw data supporting the conclusions of this article will be made available by the authors, without undue reservation.

## Ethics statement

The studies involving humans were approved by Near East University Ethical Committee and the University of Kyrenia Ethical Committee. The studies were conducted in accordance with the local legislation and institutional requirements. The participants provided their written informed consent to participate in this study.

## Author contributions

TL: Conceptualization, Formal analysis, Methodology, Writing—review & editing. CDY: Conceptualization, Data curation, Investigation, Methodology, Writing—original draft. MJMS: Writing—review & editing.
